# Enhancement of toughness and wear resistance in boron nitride nanoplatelet (BNNP) reinforced Si_3_N_4_ nanocomposites

**DOI:** 10.1038/srep27609

**Published:** 2016-06-08

**Authors:** Bin Lee, Dongju Lee, Jun Ho Lee, Ho Jin Ryu, Soon Hyung Hong

**Affiliations:** 1 Department of Materials Science and Engineering, Korea Advanced Institute of Science and Technology, 291 Daehak-ro, Yuseong-gu, Daejeon 34141, Republic of Korea; 2 Nuclear Materials Development Division, Korea Atomic Energy Research Institute, 111 Daedeok-daero 989 Beon-gil, Yuseong-gu, Daejeon 305-353, Republic of Korea; 3 Department of Nuclear and Quantum Engineering, Korea Advanced Institute of Science and Technology, 291 Daehak-ro, Yuseong-gu, Daejeon 34141, Republic of Korea

## Abstract

Ceramics have superior hardness, strength and corrosion resistance, but are also associated with poor toughness. Here, we propose the boron nitride nanoplatelet (BNNP) as a novel toughening reinforcement component to ceramics with outstanding mechanical properties and high-temperature stability. We used a planetary ball-milling process to exfoliate BNNPs in a scalable manner and functionalizes them with polystyrene sulfonate. Non-covalently functionalized BNNPs were homogeneously dispersed with Si_3_N_4_ powders using a surfactant and then consolidated by hot pressing. The fracture toughness of the BNNP/Si_3_N_4_ nanocomposite increased by as much as 24.7% with 2 vol.% of BNNPs. Furthermore, BNNPs enhanced strength (9.4%) and the tribological properties (26.7%) of the ceramic matrix. Microstructural analyzes have shown that the toughening mechanisms are combinations of the pull-out, crack bridging, branching and blunting mechanisms.

Ceramic materials have been widely used for structural applications given their superior mechanical properties (such as hardness and strength), good corrosion resistance, and stability at high temperatures. However, brittleness has remained a problem with these materials. Therefore, ceramic matrix composites (CMCs) with strong reinforcement materials have been introduced to improve the toughness of monolithic structural ceramics.

Nano-sized carbon materials such as carbon nanotubes (CNTs) and graphenes have been considered as reinforcements with outstanding mechanical properties compared to conventional fillers^1^,^2^. CNTs are utilized only at the outer surface of their structure when they are dispersed in the matrix, whereas graphenes have a two-dimensional (2-D) graphitic structure which can maximize the interfacial area in contact with the matrix^3^. Furthermore, unlike CNTs, graphene can be synthesized by facile top-down processes from graphite. Due to these advantages of graphenes, they have been widely used as a reinforcement material for CMCs^4^,^5^,^6^,^7^,^8^.

However, carbon-based nanomaterials such as CNTs and graphenes have a critical weakness when used in specific applications due to their poor high-temperature stability. CNTs and graphenes can be easily oxidized around 450–500 °C in an air atmosphere and are therefore not suitable for high-temperature applications^9^. Furthermore, the black color and the undefined cytotoxicity of carbon-based nanomaterials are serious drawbacks preventing the use of these materials as a type of reinforcement, for instance, in artificial tooth applications^10^,^11^.

To overcome the aforementioned disadvantages of carbon-based nanomaterials for CMCs, we focused on a nanomaterial based on hexagonal boron nitride (h-BN) as an alternative reinforcement for CMCs. Two-dimensional BN (BN nanoplatelet, BNNP) is a structural analogue of graphene^12^. The mechanical properties of BNNPs are comparable to those of graphenes. The elastic modulus of BNNPs ranges from 700 to 900 GPa depending on the chirality (graphenes have an elastic modulus of ~1 TPa)^13^. They also have the potential to serve as an outstanding reinforcement due to their 2D nanostructure. Moreover, unlike carbon-based nanomaterials, BNNP is chemically inert up to 950 °C^14^. Thus, BNNP-reinforced CMCs may be promising for high-temperature applications such as high-speed slide bearings, brake system components, or for certain aerospace applications such as heat shield systems for space vehicles with enhanced mechanical and thermal properties where CMCs reinforced with graphenes are degraded. Furthermore, given their additional advantage of having a white color, BNNPs have the potential to be used as a reinforcement material for ceramic composites for artificial tooth applications. Lahiri *et al.* fabricated a hydroxyapatite (HA) composite which was reinforced by BN nanotubes (BNNTs)^15^. The fabricated composite showed enhanced mechanical properties in terms of its toughness and wear-resistance properties. The BNNT-reinforced composite maintained the good biocompatibility of HA. Furthermore, Chen *et al.* reported that BNNT shows a nearly innoxious property when compared with CNTs. Although these results were obtained not with BNNP but with BNNT, through them, we anticipate that such a tendency can also be applied to BNNPs as well^16^.

Several papers have dealt with hexagonal-BN (h-BN) particles or BNNT-reinforced ceramic nanocomposites^17^,^18^,^19^,^20^; however, there are relatively few results pertaining to BNNP-reinforced ceramic nanocomposites^21^. Yue *et al.* reported the fracture toughness of BNNT- and BNNP-reinforced ZrB_2_-SiC composites. Because they used not only BNNPs but also BNNTs, investigations of the reinforcing mechanisms of 2-D nanomaterials for CMCs were not done. Furthermore, the thickness of their BNNP was 40 nm, which is quite bulky for a nanoplatelet.

In order to fabricate BNNPs, several methods also used for graphene fabrication, such as CVD^22^, solvent exfoliation^23^, micromechanical cleavage^24^ and ball-milling^25^, have been applied. Each method has its pros and cons in terms of quality and yield. For example, micromechanical cleavage using the ‘Scotch tape’ method is known for high quality and extremely low yields^24^, and CVD presents relatively high quality levels and precisely controlled thicknesses^22^. In this research, for the fabrication of BNNP-reinforced CMC, we adopt a ball-milling process; specifically, the high-energy planetary ball-milling process used for the fabrication of BNNPs. The features of the ball-milling process include a very high yield and relatively good uniformity when the process is combined with an additional centrifuging process^25^,^26^.

In this study, we fabricated a CMC reinforced only with BNNPs for the first time. For the ceramic matrix, we used silicon nitride (Si_3_N_4_), which is widely used for structural applications in harsh environments^27^,^28^,^29^. This material is applied in engine parts and in high-speed cutting tools to impart high hardness, toughness and good heat resistance. In order to enhance its performance, improvements of the tribological properties and the toughness of Si_3_N_4_-based ceramics are necessary. In addition, h-BN^30^,^31^,^32^ or graphene^33^,^34^,^35^ have been used as lubricating reinforcements of Si_3_N_4_-based ceramics. However, h-BN-reinforced Si_3_N_4_ composites are associated with the deterioration of other mechanical properties, such as the toughness and strength due to the high porosity levels caused by particularly thick h-BN reinforcement materials and the easy cleavage of the basal plane of h-BN^30^,^31^,^32^. The results with graphene/Si_3_N_4_ composites show enhanced mechanical properties, but applications of these composites are limited due to the aforementioned disadvantages of carbon-based nanomaterials. Our goal here is to replace the reinforcement with BNNP in order to solve these problems. For the homogeneous dispersion of BNNP in Si_3_N_4_ powder, BNNPs were non-covalently functionalized by polystyrene sulfonate (PSS) during the ball-milling process, and a surfactant-assisted colloidal process was adopted. The fabricated composite powders were consolidated by hot pressing (HP), and the mechanical properties of the sintered nanocomposite samples, such as the hardness, toughness and wear resistance, were characterized.

## Results

### Fabrication Process and Characterization of Non-covalently Functionalized PSS-BNNP Powders

**Figure 1:**
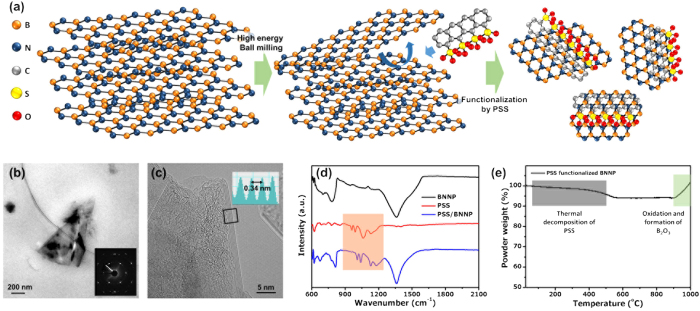
Fabrication process of BNNPs by the high-energy ball-milling process: (**a**) Schematic design of the fabrication of PSS non-covalently functionalized BNNPs by high-energy ball-milling process. (**b,c**) Low-resolution (**b**) and high-resolution (**c**) TEM images of fabricated BNNPs. (**d**) FT-IR analysis of BNNP (black), PSS (red), and PSS-functionalized BNNP (blue) powders. (**e**) TG analysis of PSS/BNNP powders.

In order to fabricate few-layered BNNPs, a planetary ball-milling process was adopted. A schematic of the fabrication process of BNNPs is illustrated in Fig. 1a. During the high-energy planetary ball-milling process, the upper layers of h-BN were detached from their bodies due to the shear force generated during the process. To prevent the restacking of exfoliated BNNPs, PSS was added as a non-covalent functionalization agent. In the solution, the dissolved PSS was combined with BNNP via π-π interaction. Without PSS, exfoliated BNNPs could easily be recombined by van der Waals force. By repeating the shear and functionalization processes during the planetary ball-milling process with a subsequent centrifuge process, BNNPs with a controlled size and thickness could be synthesized. The fabricated BNNPs have a submicron lateral size (Fig. 1b) and a thickness of less than 10 nm (Figs 1c and S1). The measured interplanar space of the synthesized BNNPs was approximately 0.34 nm, which is nearly identical to the standard interplanar distance of BNNPs^36^. Furthermore, the SAED (selected-area electron diffraction) pattern of the fabricated BNNPs presented typical six-fold symmetry along the [0001] zone axis. This result shows that the BNNPs maintain their hexagonal structure during the high-energy planetary ball-milling process. The average yield of PSS-BNNP was 17.7%, which is quite high compared to other fabrication processes such as liquid-phase exfoliation from h-BN^23^.

In order to confirm the non-covalent functionalization of BNNPs by PSS, the FT-IR spectra of the PSS, the BNNPs, and the PSS-BNNP were characterized (Fig. 1d). In the FT-IR results of the PSS and PSS-BNNP powders, the absorption peaks between 900~1200 (cm^−1^) exhibit signals which signify that the synthesized BNNPs were well functionalized by the PSS. According to the π-π intermolecular interaction with the PSS and BNNP, the peaks are relatively upshifted. The UV analysis also confirmed the existence of the characteristic peak at around 220 nm, indicating the PSS-functionalized status of BNNP (Fig. S2).

One of the considerable characteristic advantages of BNNP over graphene is high-temperature stability. A thermal gravimetric analysis of the PSS-functionalized BNNPs is shown in Fig. 1e. During the heating process from room temperature to 500 °C, the weight of the PSS-BNNP was decreased by about 5 wt.%. This result indicates that the weight percentage of the attached and functionalized PSS was approximately 5%. Furthermore, up to 900 °C, the BNNPs maintained their structure due to their superior high-temperature thermal stability. This result shows that BNNPs can be utilized for high-temperature applications. However, at temperatures above 900 °C, BNNPs are oxidized to B_2_O_3_^37^.

### Homogeneous Mixing of BNNP and Si_3_N_4_ using a Surfactant-Assisted Colloidal Process

**Figure 2:**
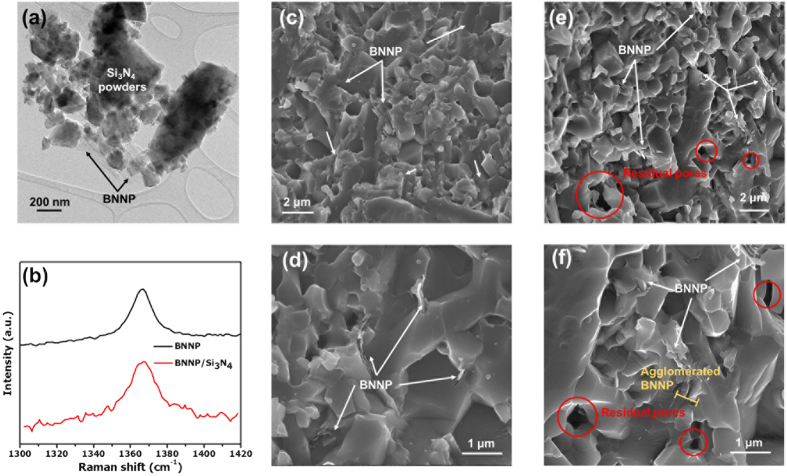
Microstructures and characterization of BNNP/Si_3_N_4_ nanocomposite powders and nanocomposites: (**a**) TEM image of the fabricated BNNP/Si_3_N_4_ nanocomposite powders. (**b**) Raman analysis of BNNP and consolidated BNNP/Si_3_N_4_ nanocomposites. (**c–f**) Microstructures of the fracture surface of the BNNP/Si_3_N_4_ nanocomposites. (**c,d**) Low- and high-resolution SEM images of the 1 vol.% BNNP/Si_3_N_4_ nanocomposite. (**e,f**) Low- and high-resolution SEM images of the 3 vol.% BNNP/Si_3_N_4_ nanocomposite.

Submicron Si_3_N_4_ powders were used, with 5 wt.% of Y_2_O_3_ and 2 wt.% of Al_2_O_3_ powders mixed with Si_3_N_4_ to increase the sinterability. The powders were mixed by tumbler ball-milling to ensure a homogeneous mixing. We then adopted a surfactant-assisted colloidal process to obtain homogenously dispersed BNNPs in the Si_3_N_4_ matrix. First, PSS-functionalized BNNPs at various amounts were dispersed in D.I. water by ultrasonication. The Zeta potential of the solution was −41.44 mV for a 0.1 mg/ml concentration. On the other hand, Si_3_N_4_ powder was dispersed in D.I. water with CTAB, a well-known cationic surfactant^33^. Because the PSS-BNNP powders were negatively charged, in order to maximize the dispersion stability of the Si_3_N_4_ powder, the surface of the Si_3_N_4_ powder must be modified so that the polarity is more positive. Figure S3 shows the Zeta potential difference between the PSS-BNNPs and the CTAB-Si_3_N_4_, as measured when changing the amount of CTAB to optimize the CTAB-to-Si_3_N_4_ ratio. With 0.1 wt.% of CTAB, the CTAB-Si_3_N_4_ powders have the highest Zeta potential value of 80.12 mV. The opposite charged their Zeta potential values imply that BNNPs and Si_3_N_4_ can attach to each other in a solution to achieve a homogeneous dispersion of BNNPs in the Si_3_N_4_ powder^38^. The microstructures of the dried and calcined powder showed that the BNNPs were dispersed homogeneously between the Si_3_N_4_ particles (Figs 2a and S4a,b).

### Consolidation and Microstructures of BNNP/Si_3_N_4_

After the calcination process, the PSS and CTAB-removed BNNP/Si_3_N_4_ composite powders were consolidated using a hot-pressing (HP) process in a nitrogen atmosphere. The sintering schedule of the hot-pressing process is shown in Fig. S5a. The sintered materials showed relatively high densification (over 99.7%; the density of BNNP is assumed to be 2.1 g/cm^3^ based on the density of bulk h-BN) until the volume content of the BNNP was increased to 2 vol.%. At 3 vol.% of BNNP-reinforced Si_3_N_4_, the relative density decreased (98.6%, Fig. S5b). In order to identify the phase of the samples after the consolidation process, XRD and Raman spectroscopy analyses were conducted. An XRD analysis of the hot-pressed BNNP/Si_3_N_4_ composite shows that nearly all phases of Si_3_N_4_ were transformed into the β-phase (Fig. S6). Due to the low content of BNNP, no characteristic diffraction peak of BN could be observed. However, from the Raman analysis of a sintered sample of BNNP/Si_3_N_4_ (Fig. 2b), a clear peak (the lower red line in the graph) at around 1365 cm^−1^ was detected^39^,^40^. We can confirm that the BNNP survived the high-temperature sintering process. The microstructures of a fracture surface of the 2 vol.% BNNP/Si_3_N_4_ sample show the dispersion status of the BNNPs in the Si_3_N_4_ matrix (Fig. 2c,d). The BNNPs were homogeneously dispersed in the Si_3_N_4_ matrix, and the fracture surfaces consisted of both intergranular and transgranular surfaces. However, when the volume contents of BNNP were increased to 3 vol.%, the microstructures changed significantly. As shown in Fig. 2e,f, the BNNPs in the matrix in this case were more agglomerated, and the average agglomerated BNNP cluster size was increased (Fig. S7). Agglomerated BNNPs can disturb the consolidation of the Si_3_N_4_ matrix during the hot-pressing step, causing more residual pores in the composite after sintering. Several pores originated from the stacked BNNPs in the Si_3_N_4_ matrix. An excess amount of BNNP provides defect sites in the matrix, which can bring about cracks from the inside of the material. Similarly, several theoretical and experimental results were reported with graphene, demonstrating that the modulus and hardness of graphene are sensitive to the total number of layers in the graphene^41^,^42^. It can also be deduced that the mechanical properties of the agglomerated BNNPs were reduced, having an adverse effect, as observed with the 3 vol.% content of the BNNPs in the composite.

BNNP/Si_3_N_4_ composites were polished and etched in order to determine the grain size variation with the amount of BNNPs in the Si_3_N_4_ composites. From the SEM analysis of the surfaces, a bimodal distribution of the grain size is observed, and pores were detected with the increment of the BNNP content (Fig. S8). Average grain sizes and the average aspect ratio of the grains were calculated from the results of an image analysis (Fig. S9), and the variation of the grain sizes did not show any noticeable tendency. However, the average aspect ratio increased with the addition of BNNPs. appeared that the dispersed BNNPs provided the direction for the anisotropic grain growth of Si_3_N_4_ grains, after which the aspect ratio of the Si_3_N_4_ have been increased. The increased aspect ratio of the Si_3_N_4_ grains represents an effective self-grain load transfer mechanism in Si_3_N_4_-based composites.

### Mechanical Property Characterization of BNNP/Si_3_N_4_

**Figure 3:**
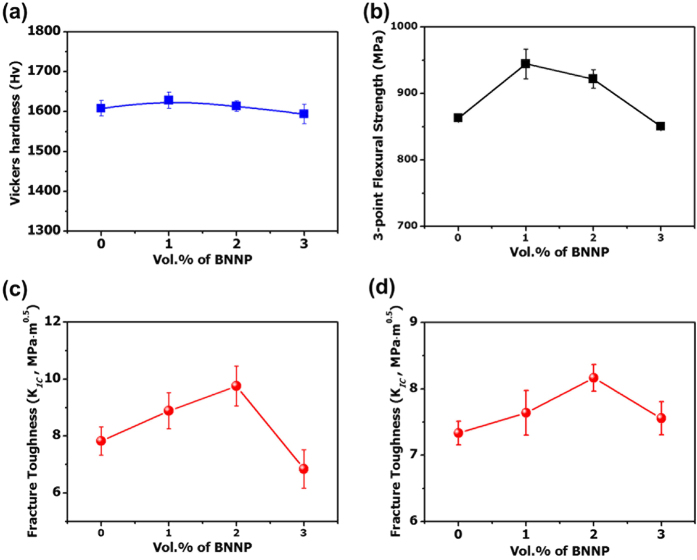
Characterization of the mechanical properties of BNNP/Si_3_N_4_: (**a**) Vickers hardness analysis (**b**) three-point flexural strength (**c**) fracture toughness according to a Vickers indentation fracture test, and (**d**) fracture toughness as measured by the single-edge notched beam method.

To measure the mechanical properties of the BNNP/Si_3_N_4_ composites, Vickers hardness tests, three-point bending tests, and fracture toughness tests using the single-edge notched beam method (SENB) were conducted. No significant enhancement of the Vickers hardness values was noted (Fig. 3a). In order to compare the hardness value with other results, the hardness values of a graphene-like 2D nanomaterial-reinforced CMC are listed in Table S25^8^,^33^,^34^,^43^,^44^,^45^,^46^,^47^,^48^,^49^,^50^,^51^,^52^,^53^,^54^. Although no BNNP/ceramic results are given, the two samples show a consistent tendency in terms of their hardness levels. For ceramic matrices which have relatively low initial hardness, such as hydroxyapatite^43^,^48^, SiO_2_^51^, and CaSiO_3_^50^, their composites show enhanced hardness results with the addition of graphene. However, for Si_3_N_4_^33^,^34^,^49^, Al_2_O_3_^5^,^8^, ZrO_2_^52^, and ZrB_2_^45^, which can be classified as hard ceramics, their hardness levels after the formation of the composites decreased. For the “soft” ceramic matrix listed above, graphene played a greater role in the increase in hardness.

Figure 3b shows the three-point bending test result of 1 vol.% of BNNP/Si_3_N_4_ which showed increase in bending strength from 863 ± 4.0 MPa to 944 ± 22.5 MPa, corresponding to 9.4% increase in comparison to pure Si_3_N_4_. When nano-sized and low-dimensional reinforcements are introduced, a homogeneous dispersion of the reinforcements is an important issue for enhanced properties of the composites. Several results which deal with graphene-reinforced CMC show decreased bending strengths^34^,^49^. Residual pores due to the agglomeration of graphene can result in the formation of a submicron-sized notch. Only results which resolve the dispersion problems of graphene can enhance the strength of the resulting composites. Similar problems can occur in BNNP/ceramic systems; therefore the functionalization of BNNP with PSS and the synergetic dispersion with the cationic surfactant CTAB are used in our system in order to prevent the agglomeration of BNNP. BNNPs which are homogeneously dispersed by a surfactant provide effective load transfer capabilities between the BNNPs and the matrix in the composite system. Pull-out phenomena were also observed at the fracture surfaces (Fig. 2d,f). However, as the amount of BNNP increases, the tendency to aggregate at the grain boundaries arises, resulting in residual pores (Fig. 2f). The relative density also decreases with an increase in the number of internal pores caused by the agglomeration of BNNPs. These residual pores act as crack-initiation points; therefore, with more than 3 vol.% of BNNP added, the three-point flexural strength decreases.

The fracture toughness levels of the composites were measured by the Vickers indentation fracture (VIF) test and by the SENB fracture toughness measuring method. Although the VIF method has several limits when used to measure the intrinsic K_I*C*_ value^55^, it is still a useful and convenient method to estimate the resistance to crack propagation. For the SENB method, we cannot prepare identical dimensions for our test specimen with ASTM due to the limitation of the consolidation method used and the after-treatment technique^56^,^57^, but the result is valid nonetheless to determine the tendency of the fracture toughness of BNNP/Si_3_N_4_ nanocomposites depending on the amount of BNNP. Fortunately, the fracture toughness levels as measured by those two testing methods were nearly identical. The VIF results calculated from the crack length as induced by Vickers indentation indicate that the toughness was increased by 24.7% in case of 2 vol.% BNNP-reinforcement composite (from 7.83 ± 0.5 MPa·m^0.5^ to 9.76 ± 0.7 MPa·m^0.5^, Fig. 3c). The fracture toughness from the SENB test also showed a 12.7% increase from 7.33 ± 0.18 MPa·m^0.5^ to 8.17 ± 0.2 MPa·m^0.5^ (Fig. 3d) at 2 vol.% of BNNP reinforced. We expected that the fracture toughness would increase because the planer-type 2D BNNP may provide toughening effects as a fiber-type reinforcement material does in the brittle solids. Dispersed or aligned fibers in brittle matrix composites can absorb the energy of crack propagation via several mechanisms. Furthermore, BNNP can maximize each toughening mechanism due to its unique 2D structure. A detailed explanation of the advantages of 2D nano-reinforcements is described below.

**Figure 4:**
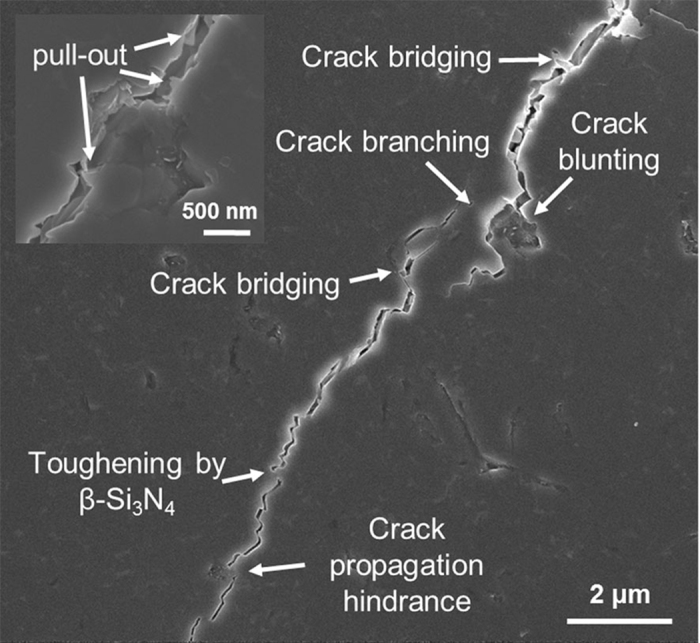
Summarized toughening mechanisms of the BNNP in the Si_3_N_4_ matrix. These include nanoplatelet pull-out, crack bridging, branching, and blunting. The inset image represents nanoplatelet pull-out, which is the most frequent mechanism.

Figure 4 summarizes several examples of the microstructural evidence of toughening by BNNP. Homogeneously dispersed BNNPs absorb the crack propagation energy via various mechanisms. The nanosheet pull-out mechanism (or debonding) was the most frequently noted toughening mechanism. Although we did not apply a treatment to enhance the interfacial property between the BNNP and the matrix, considerable pull-out phenomena can dissipate the crack propagation energy. BNNP may act as a bridging ligament in the Si_3_N_4_-based composite. BNNP is too rigid to be broken by internal cracks in the composite; therefore, BNNP can bridge the cracks and absorb the energy for crack propagation. Several crack bridging phenomena have been developed at the final stage of the rupture. Because the lateral size of the BNNP is approximately 150~500 nm, a crack with a width within this range can be bridged by dispersed BNNPs. A homogeneous dispersion of BNNPs can also cause crack branching, deflection, and crack blunting. Several microstructures represent evidence of crack branching and deflection in Si_3_N_4_ without BNNP. When the composites contain a high content of BNNP (over 3 vol.%), agglomeration problems can occur. Residual pores caused by the agglomeration of BNNPs become a good pathway for crack propagation; therefore, when the volume content of BNNP increases to 3 vol.%, the toughness decreases. The high-resolution TEM analysis results shown in Fig. S11 clearly demonstrate the detriment of agglomerated BNNP in high-volume-ratio composites. These results show the junction area of Si_3_N_4_ and two agglomerated BNNPS. An increase in the BNNP ratio enhances the agglomeration of the BNNPs, resulting in a thickness which exceeds 100 nm. Moreover, saturated BNNP jumbled at the boundary can occasionally inhibit full densification. Furthermore, the amorphous phase can be detected in the triple junction of Si_3_N_4_ and in two BNNP areas. The small red circle in Fig. S11(a) indicates the area demarcated in Fig. S11(b). It appears that the agglomerated BNNPs form an intergranular amorphous pocket at the grain boundary. From the result of Wei *et al.*^58^, who analyzed the amorphous phase in h-BN/Si_3_N_4_ composites with Y_2_O_3_ and Al_2_O_3_ sintering additives, the amorphous phase mainly contains Y and Si and a small amount of Al. They concluded that the chemical stability of h-BN in h-BN/Si_3_N_4_ ceramic composites is quite good; therefore, the phase composition of the amorphous phase in the Si_3_N_4_ ceramic matrix is unaffected by the addition of h-BN. Those nano-sized intergranular amorphous phases can deteriorate the composites^59^, representing another reason why the mechanical properties decreased with high amounts of BNNPs.

### Wear resistance of BNNP/Si_3_N_4_

**Figure 5:**
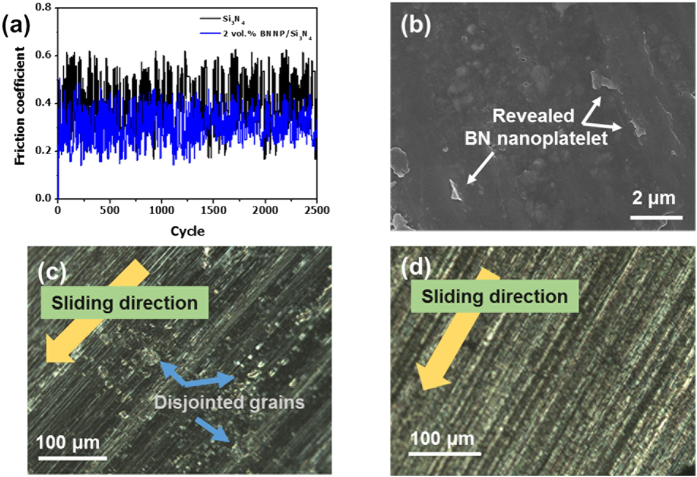
Wear resistance of the BNNP/Si_3_N_4_ nanocomposite: (**a**) Friction coefficient of monolithic Si_3_N_4_ (black) and 2 vol.% BNNP/Si_3_N_4_ (blue) samples. (**b**) SEM analysis of the worn surface of the BNNP/Si_3_N_4_ nanocomposite. BNNPs are clearly out of the surface of the composite. (**c**) OM image of the worn surface of the monolithic Si_3_N_4_ and (**d**) the 2 vol.% BNNP/Si_3_N_4_ nanocomposites. Nearly unnoticeable grain pull-out occurred during the wear test.

In order to measure the self-lubricating property of BNNP, wear-resistance tests with an alumina counterpart were undertaken. The BNNP-reinforced Si_3_N_4_ was found to enhance not only the strength and toughness but also the wear resistance. Even under a harsh normal load (load: 39.2 N, sliding speed: 100 rpm), the composites were barely worn away such that the wear loss could not be surveyed. The average friction coefficient (or coefficient of friction, COF) decreased from 0.38 to 0.30 with 2 vol.% of dispersed BNNP (Fig. 5a). However, for the 3 vol.% BNNP/Si_3_N_4_ composite, the COF was 0.42, a slightly increased value, indicating that the tribological properties were degraded. The observation of the wear track was made with SEM and optical microscopy. In the SEM image of the worn surface shown in Fig. 5b, BNNPs were exposed on the worn surface of the BNNP/Si_3_N_4_ composites. For the monolithic Si_3_N_4_ sample, the optical microscopy analysis results showed that some grains had fallen out during the test (Fig. 5c); however, nearly unnoticeable grain pull-out phenomena during wear test were detected in the BNNP/Si_3_N_4_ composite (Fig. 5d). It appears therefore that the exposed BNNP during the friction test acts as a protection barrier, preventing the dismounting of the grains.

## Discussion

### Toughening Mechanisms by BNNP in a Ceramic Matrix

**Figure 6:**
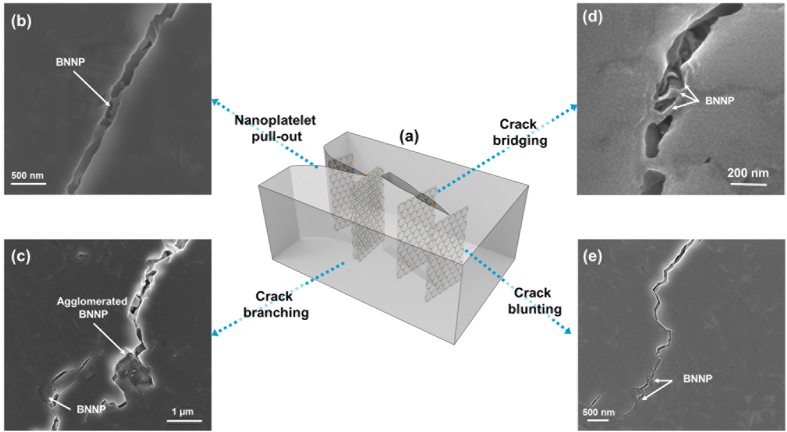
Schematic image and microstructural evidence of the toughening mechanisms in the ceramic matrix: (**a**) Schematic image, (**b**) nanoplatelet pull-out, (**c**) crack branching, (**d**) crack bridging, and (**e**) crack blunting.

The microstructurally observed toughening mechanisms are summarized in Fig. 6 with a schematic illustration. In this case, pull-out, crack branching, crack bridging, and crack blunting phenomena occurred in the BNNP/Si_3_N_4_ composites (Fig. 6). As noted above, the most dominant toughening mechanism was the pull-out phenomenon. Unlike typical long fiber-reinforced ceramic matrix composites, the Young’s modulus of BNNP is too high as compared to the interfacial interaction between the BNNP and the Si_3_N_4_ to be broken due to crack propagation. Therefore, there were no BNNP ruptures, but the debonding of BNNP with the surrounding Si_3_N_4_ and pull-out occurred. Many studies have investigated toughening mechanisms and devised mathematical expressions for fiber-reinforced ceramic matrixes. However, only a few have studied nanosheet reinforcement in CMCs^60^. According to Chawla^61^, Δ*G*_*c*(*fiber*)_, the toughness variation is derived during the fiber pull-out process,


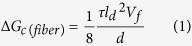


where *τ* is the interface friction stress, *l*_*d*_ is the fiber length, *V*_*f*_ is the fiber volume fraction, and d is the diameter of the fibers. This equation is simplified with several assumptions, such as the exclusion of the fiber fracture energy and an average pull-out length of approximately (*l*_*d*_)/(4).

In order to simplify further the calculation of the pull-out behavior in the BNNP/Si_3_N_4_ composite system, we modified the equation by considering the reinforcement from the fiber to the nanoplatelet, assuming that the width of the nanoplatelet was w and that t denotes the thickness of the nanoplatelet, corresponding to the diameter of the fiber. In addition, *l*_*d*_ is the length of the nanoplatelet. A schematic of the nanoplatelet pull-out behavior is illustrated in Fig. S12. The pull-out work for such a nanoplatelet is denoted as follows:


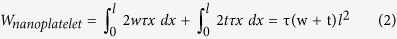


Under the assumption also used with the fiber pull-out system (*W*_*fiber*_ = (1)/(2)*τπdl*^2^), the average length of the nanoplatelet pull-out is approximately (*l*_*d*_)/(4); therefore, the work from the nanoplatelet is 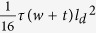. Because the nanoplatelet cross-sectional area is wt, 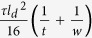 is the nanoplatelet pull-out work per unit area of the reinforcement. Through multiplication with the volume fraction of the nanoplatelet, *V*_*f*_, the change in the toughness during the nanosheet pull-out process can be derived as


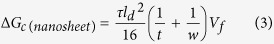


Assuming that the nanoplatelet is very thin and square-shaped, then t ≪ w, and *l*_*d*_ = *w*. The aspect ratio (AR) of the nanoplatelet can then be defined as *w*^2^/t.


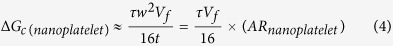


Further modification of the equation is necessary because we assume the interfacial stress τ to be constant, whereas it scales with the bond stress and changes during the pull-out process. We assume that the pull-out direction of the nanoplatelet is only longitudinal, and we disregarded the flexibility of the BNNP. Nevertheless, we can determine the tendency of the energy dissipation during the pull-out process with dimensions of the nanoplatelet. In order to maximize of the toughening of the BNNP, the BNNP should be relatively thin with a long length so as to retain a long path for the pull-out process. Moreover, for the square-type nanoplatelet, a large aspect ratio of the nanoplatelet is important to optimize the pull-out efficiency of the nanoplatelet.

The formula shows that an increase in *V*_*f*_ can also increase the toughness of the CMC which is reinforced by 2D nanoplatelets. With up to 2 vol.% of BNNP/Si_3_N_4_ nanocomposites, it appears that the BNNPs maintain their dispersion stability in the matrix. From Fig. 3c, the first three points show an almost linear tendency with an increase in the BNNP content. However, when the volume ratio of the BNNP exceeds 3 vol.%, the fracture toughness is reduced, which differs from the expectations. Initially, the formula is based on the friction force between the outer surfaces of the nanoplatelets. However, nanoplatelets tend to agglomerate over a specific content (in this research, the 3 vol.% BNNP/Si_3_N_4_ composite shows this tendency first), and agglomerated nanoplatelets show a reduced aspect ratio, which also can diminish the pull-out energy. Furthermore, as noted above, agglomerated nanoplatelets induce flaws such as pores or are typically located at the grain boundary instead of inside the grain, where they may promote crack propagation. Hence, when the nanoplatelets exceed a certain amount, the formula cannot always be applied given the agglomeration of the nanoplatelets due to their strong van der Waals force.

### Wear-Resistance Mechanism in BNNP-Reinforced Si_3_N_4_ composites

The wear resistance of the BNNP/Si_3_N_4_ composite shows an increase compared to that of the monolithic Si_3_N_4_. Although the mass loss during the friction tests could not be measured because the Si_3_N_4_ and BNNP/Si_3_N_4_ samples were very small, distinct decreases of the COF were noted after the addition of 2 vol.% of BNNP. From the microstructural analysis of the wear track of the monolithic Si_3_N_4_ and BNNP/Si_3_N_4_ samples (Fig. 5c,d), significant differences between the two samples were noted. Because there is no discussion of lubricating mechanisms of BNNPs in bulk materials, we refer to hypotheses about CNT- or graphene-reinforced composite materials. Puchy *et al.* proposed wear-resistance mechanisms which apply to the CNT-reinforced Al_2_O_3_ system^62^. During wear test, wear debris, which is a mixture of alumina and CNT particles, forms a ‘lubricating film’ on the worn surface, which enhances the wear properties of the matrix. Wenzheng *et al.* also discussed how refined GNP creates a protective layer which protects against abrasion in GNP/Ni_3_Al composites^63^.

**Figure 7:**
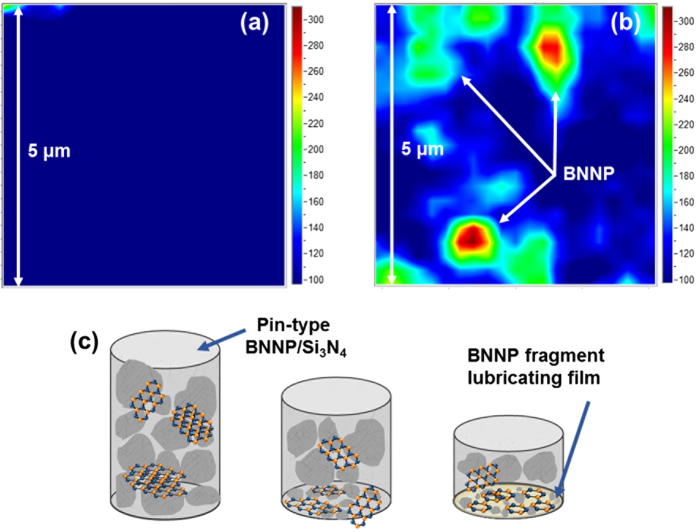
Wear-resistance mechanism of BNNPs in the ceramic matrix: (**a,b**) show the Raman spectroscopy mapping result at 1365 cm-1 for the BNNP/Si_3_N_4_ nanocomposites (**a**) before the friction test and (**b**) after the friction test. (**c**) Suggested lubricating mechanism of BNNP in the ceramic matrix.

In the BNNP/Si_3_N_4_ composite system, we assume that similar mechanisms operate during friction tests. Two steps of the lubricating process are proposed; one is the direct protection of covered BNNP and the other is the formation of a protection layer composed of fragments of BNNP. Raman-mapping analysis results of the BNNP/Si_3_N_4_ composite before and after the friction test are shown in Fig. 7a,b, respectively. Through this analysis, the existence of BNNP on the surface can be identified. Moreover, the color and scale in Fig. 7a,b indicate the intensity (or count) from the specific Raman peak number. Before the friction test, BNNPs are scarcely visible on the surface of the composite. However, after the experiment, a BNNP peak at around 1365 cm^−1^ was noted within the same scale range of monolithic Si_3_N_4_. The red region in the figures indicates that the BNNPs cover the composite, protecting it from friction. BNNPs were exposed on the surface during the friction test, showing a strong Raman peak. The wide green area shows that the BNNP protection area which is composed of frittered BNNP during the test. We consider that these two mechanisms can enhance the wear resistance of the composite. The lubricating mechanisms of the BNNP/Si_3_N_4_ composites are simply expressed in Fig. 7c. However, when more than 3 vol.% of BNNP is added to the composite, the COF increases. Degradation of the sinterability due to the agglomerated BNNPs weakens the grain boundary. Although the BNNPs offer protection by forming a lubricating film on the surface of the composite, the weakened grain boundary easily pulled out, likely causing friction on the surface of the composite.

In summary, for the first time developed a BNNP-reinforced Si_3_N_4_ matrix composite which possesses enhanced mechanical properties was reported. Mass production of non-covalently functionalized BNNPs was established by a wet ball-milling process. The fabricated composite demonstrated enhanced strength (9.4%), toughness (24.7%), and wear resistance (a 26.7% decrease of the COF). Nanoplatelet pull-out, crack bridging, branching, and deflection occurred during crack propagation. Above all, pull-out is considered to be the most frequent toughening mechanism in BNNP-reinforced composites. Lubricating mechanisms of BNNPs in ceramic matrices were proposed in this study. The first such mechanism involves the covering of ceramic grains by a nanosheet, and the second is a nanosheet fragment which forms a lubricating film on the friction surface of the composite. We discovered the potential of BNNPs as a reinforcement material for ceramic matrix composites which can be utilized as a filler in high-temperature applications such as aerospace applications and brake systems due to their stability at high temperatures.

## Methods

### Fabrication of PSS-functionalized BNNP powders

A horizontal planetary mill (Fritsch Pulverisette 5) was used for the exfoliation of h-BN. Then, 1.5 g of h-BN powder (3~10 μm, Kojundo Korea Co., Ltd., used as received) and 1.5 g of PSS (Mw ~ 70000, Sigma-Aldrich, used as received) were added to a stainless steel grinding bowl. The diameter of the stainless steel balls used in the planetary mill was 10.3 mm and the milling condition was 150 rpm for 24 hours. The ball-to-powder ratio was 50:1, and 15 ml of IPA was used as a solvent. After the planetary ball-milling process, the milled product was collected and an additional 500 ml of IPA was added, followed by sonication for 1 hour. The dispersed PSS-functionalized BN solution was centrifuged at 2000 rpm for 20 minutes to remove the aggregated BN and thick sheets. The supernatant of the centrifuged solution was collected by a filtering process with a nylon membrane filter with a 0.2 μm pore size. During the filtering process, BNNPs were washed with 500 ml 10% of a HCl solution which was diluted with D.I. water three times in order to remove residual contaminants such as Fe^3+^ during the planetary ball-milling process. They were subsequently re-washed with D.I. water. The washed BNNPs were subsequently dried at 80 °C for one day.

### Composite powders and composite preparation

Submicron-sized 92 wt.% Si_3_N_4_ powders (Kojundo Korea Co., Ltd, used as received), 5 wt.% of Y_2_O_3_ (Sigma-Aldrich, used as received), and 2 wt.% of Al_2_O_3_ (Sigma-Aldrich, used as received) were mixed with a tumbler ball-milling process in ethanol (12 mm diameter Si_3_N_4_ balls, with a ball-to-powder ratio of 20:1, 200 rpm and 12 hours). The mixed powders were dried and sieved through a 125-μm mesh and subsequently dispersed by ultrasonication for 1 hour in D.I. water with various amounts of CTAB (Sigma-Aldrich, used as received). Various amounts of the previously produced PSS-functionalized BNNP powders were also dispersed in D.I water by ultrasonication for 1 hour at a concentration of 0.5 mg/ml. The two solutions were combined and ultrasonicated for an additional hour, and the D.I. water was then evaporated while stirring. After processing, the dried composite powders were heat-treated at 550 °C for 1 hour to remove the surfactants (CTAB) and the functional agents (PSS). The heat-treated powders were ground and sieved through a 125-μm mesh. The composite powders were loaded into a graphite mold with a diameter of 20 mm. Hot pressing was performed in a nitrogen atmosphere at 1750 °C for 2 hours under a pressure of 40 MPa. The heating schedule of the hot-pressing process is shown in Fig. S5. The sintered BNNP/Si_3_N_4_ composites had a cylindrical shape with a 20 mm diameter and a height of 2 mm.

### Characterization of the powders and composites

Microstructural analyses of the PSS-functionalized BNNP, BNNP/Si_3_N_4_ composite powders and composites were conducted with a scanning electron microscope (Hitachi S-4800, Nova 230), a transmission electron microscope (Tecnai G2 F39 S-Twin) and a scanning probe microscope (Park Systems, XE-100). FT-IR spectra were characterized by the attenuated total reflectance (ATR) method (Jasco FT/IR-4100 type-A spectrometer); the XRD were analyzed using a D/MAX-lllC analyzer (3 kW). Thermal gravimetric analyses were conducted using a Setsys 16/18 device (Setaram). Raman spectroscopy was analyzed using a high-resolution dispersive Raman microscope (LabRAM HR UV/Vis/NIR, excitation at 514 nm).

For the surface analysis, the composites were ground, polished and plasma-etched. Polishing processes were performed with the standard technique down to a diamond and silica size of 0.25 μm. Samples were etched using a reactive ion etching (RIE) system (power = 200 W, pressure = 10 mTorr, O_2_ flow = 30 sccm, CF_4_ flow = 60 sccm, and time = 5 min). Etched surfaces were measured by SEM, and image analyses were done with software (ImagePartner, SARAMSOFT).

The mechanical properties of the Si_3_N_4_ and BNNP/Si_3_N_4_ composites were also analyzed. The hardness of the BNNP-reinforced Si_3_N_4_ composites was measured using the Vickers indentation method under a load of 19.6 N and with a loading time of 10 seconds (HM-124). The hardness value (H) was estimated by the following equation,


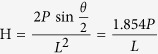


where P is the load (N), θ is the angle between opposite faces of the diamond tip (=136°), and L is the length of the indentation diagonals (m). Ten different measurements were conducted for each type of Si_3_N_4_-based composite.

The fracture toughness of the composites was estimated by two methods. The first involved measuring the lengths of the cracks caused by Vickers indentation via the following equation,


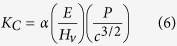


where E is Young’s modulus (here, we used a value of 330 GPa), H_v_ is the Vickers hardness (as measured), with P as the applied load, 19.6N in this case), and c is the length of the crack as measured from the center of the indentation. α is a calibration constant; in this research, a value of 0.016 was used. Before we measured the Vickers hardness of the nanocomposites and measured the lengths of the cracks by the SEM analysis, at least 20 cracks were measured to calculate the average fracture toughness of the composites.

The second method used to characterize the fracture toughness is the SENB (single-edge notched beam) method corresponding to ASTM C1421. In this research, specimens could not have identical dimensions due to the limited sample preparation methods and the post-treatment techniques used. Five specimens for each type of Si_3_N_4_-based composite were tested under three-point bending with an Instron 5583 device at a speed of 0.2 mm/min. The specimens were 11 mm×2.7 mm×2 mm in size. The fracture toughness values according to the SENB method were calculated using the following equation:


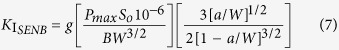


Here, *P*_*max*_ is the maximum force, *S*_*o*_ is the span length, and B and W are the width and thickness of the specimen, respectively. Additionally, a is the crack depth of the specimen. Here, g is calculated as follows:


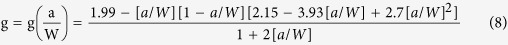


The notch of the samples was created with a razor blade, and the depth of each notch was 0.15 mm. The thickness of the notch was measured and found to be approximately 0.25 mm. A schematic illustration and the microstructure of the machined notch on the sample are shown in Fig. S11.

A three-point bending test was conducted using an Instron 5583 machine with a crosshead speed of 0.2 mm/min. Based on ASTM 1161-02c, the dimensions of the BNNP/Si_3_N_4_ composite specimens were 12.38 mm×2.75 mm×2.06 mm, which is proportional to the ASTM standard. The strength was then calculated using the following equation,


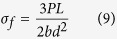


where P is the fracture load as determined from the bending test, L is the support span length, b is the thickness of the specimen, and d is the width of the samples. Five specimens for each type of Si_3_N_4_-based composite were tested.

The wear resistance behavior of the BNNP-reinforced Si_3_N_4_ composites was measured in unlubricated pin-on-disk experiments against a polished commercial Al_2_O_3_ disk. Si_3_N_4_ composites were manufactured at 1.5 φ and with a 7 mm cylindrical shape. The applied load was 39.2N, the rotational speed was 100 rpm, and 2500 cycles were run. The friction coefficients were constantly recorded during the tests. After a tribological test, the worn surfaces of the Si_3_N_4_ composites were characterized by optical microscopy, SEM, and RAMAN spectroscopy.

## Additional Information

**How to cite this article**: Lee, B. *et al.* Enhancement of toughness and wear resistance in boron nitride nanoplatelet (BNNP) reinforced Si_3_N_4_ nanocomposites. *Sci. Rep.* **6**, 27609; doi: 10.1038/srep27609 (2016).

## Supplementary Material

Supplementary Information
