# Supplementary material for: Enhancement of toughness and wear resistance in boron nitride nanoplatelet (BNNP) reinforced Si3N4 nanocomposites
Source: Sci Rep. 2016 Jun 8;6:27609. doi: 10.1038/srep27609 (PMC4897790; doi:10.1038/srep27609)
Supplement: Supplementary Information [file srep27609-s1.doc]

**Supporting Information**

**Enhancement of toughness and wear resistance in boron nitride nanoplatelet (BNNP) reinforced Si_3_N_4_ nanocomposites**

Bin Lee^1^, Dongju Lee^2^, Jun Ho Lee^1^, Ho Jin Ryu1,3,*[[1]](#footnote-2)), and Soon H. Hong1,*[[2]](#footnote-3))

Mr. Bin Lee, Mr. Jun Ho Lee, Prof. Ho Jin Ryu^*^, and Prof. Soon Hyung Hong^*^

Department of Material Science and Engineering,

Korea Advanced Institute of Science and Technology (KAIST),

291 Daehak-ro, Yuseong-gu, Daejeon 34141, (Republic of Korea)

Dr. Dongju Lee

Nuclear Materials Development Division, Korea Atomic Energy Research Institute, 111 Daedeok-daero 989 Beon-gil, Yuseong-gu, Daejeon 305-353, (Republic of Korea)

Prof. Ho Jin Ryu^*^

Department of Nuclear and Quantum Engineering,

Korea Advanced Institute of Science and Technology,

291 Daehak-ro, Yuseong-gu, Daejeon 34141, (Republic of Korea)


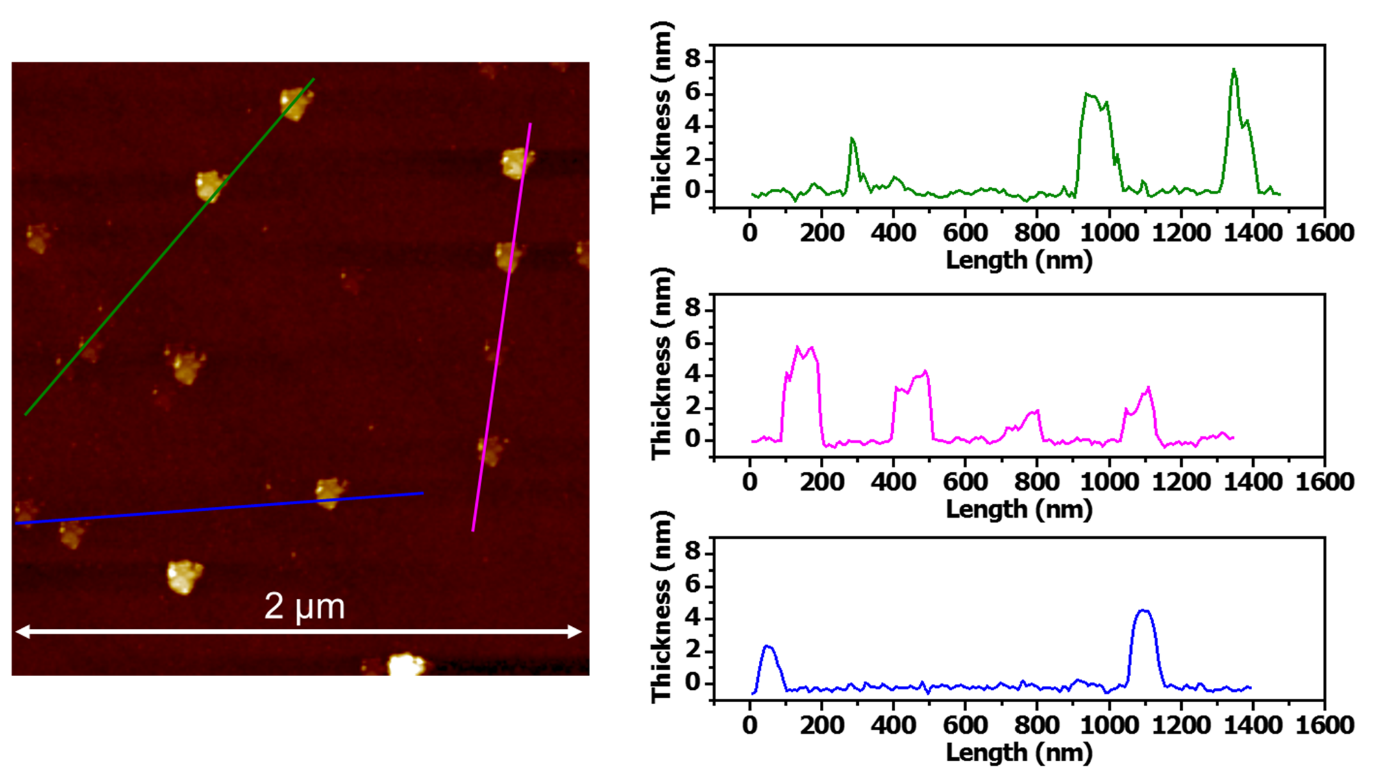
**Supplementary Figure 1. *AFM analysis result of PSS functionalized BNNP by planetary ball-milling process.*** BNNPs have a submicron lateral size and a thickness of around 5 nm.


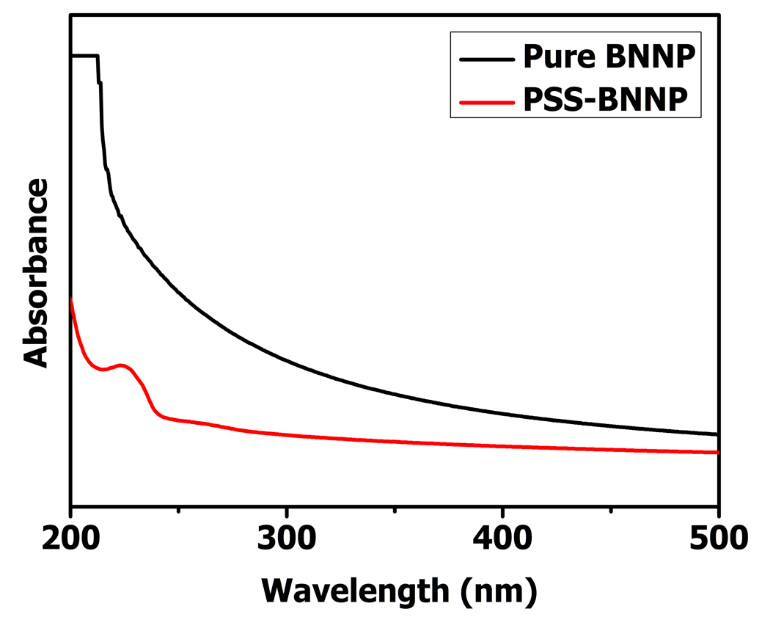


**Supplementary Figure 2. *UV analysis result of PSS-non-covalently functionalized BNNP and non-treated BNNP.*** Those two solutions are dispersed in IPA. The characteristic around 220 nm indicates the PSS-functionalized status of BNNP


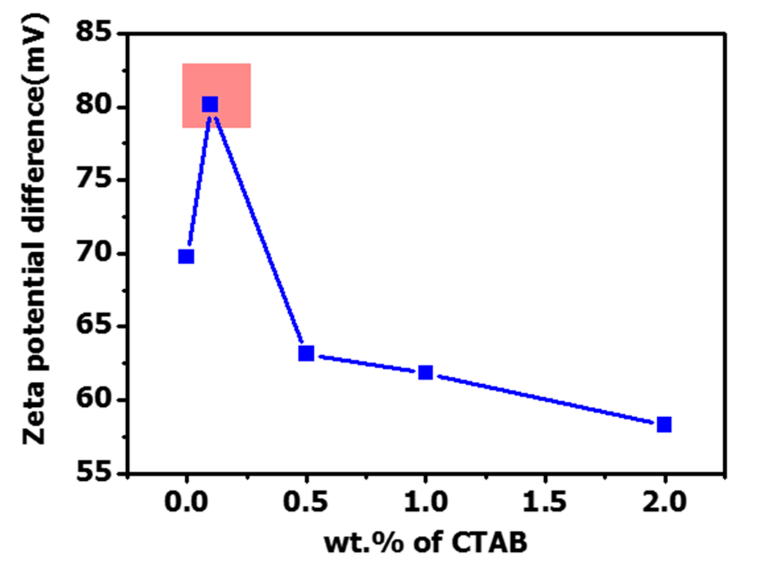


**Supplementary Figure 3. *Zeta potential difference between PSS-BNNP and CTAB-Si_3_N_4_***. 0.1 wt.% of CTAB is the optimum amount in order to homogeneous mixing of BNNP and Si_3_N_4_ powders.


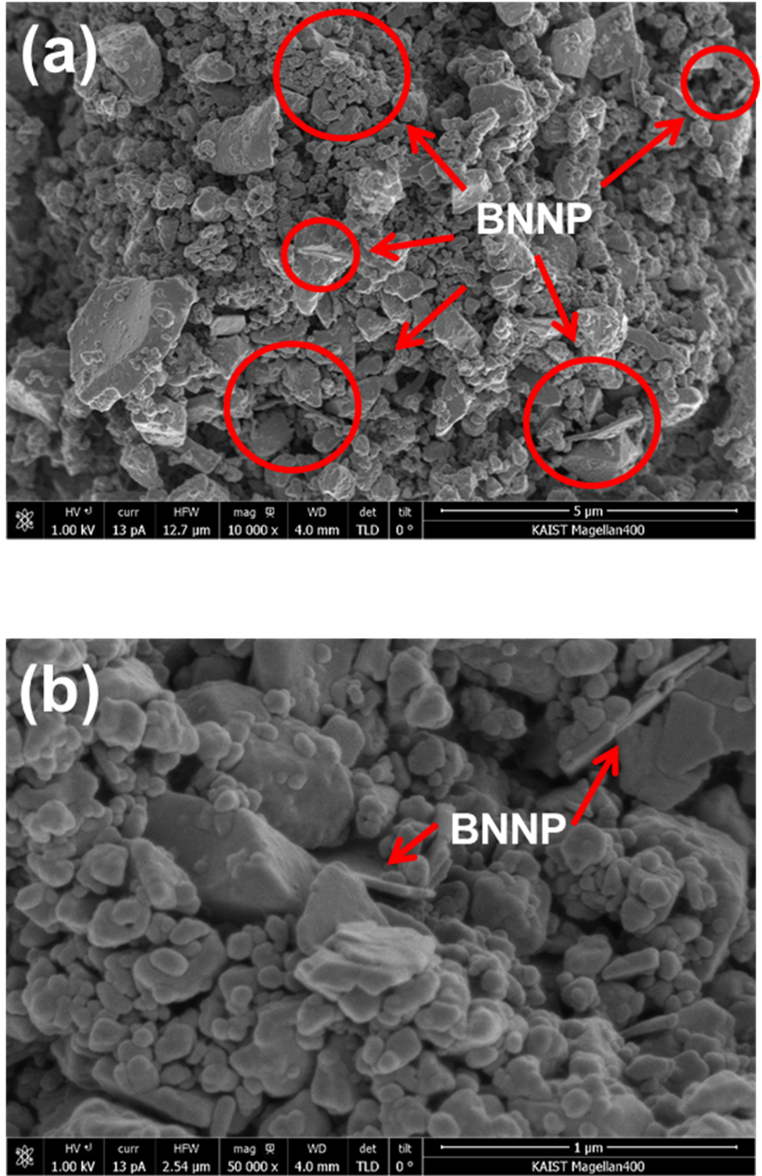


**Supplementary Figure 4. *SEM images of BNNP/Si_3_N_4_ nanocomposite powders in (a) low and (b) high resolution.*** Due to PSS and CTAB, BNNPs are homogeneously dispersed in Si_3_N_4_ powders.


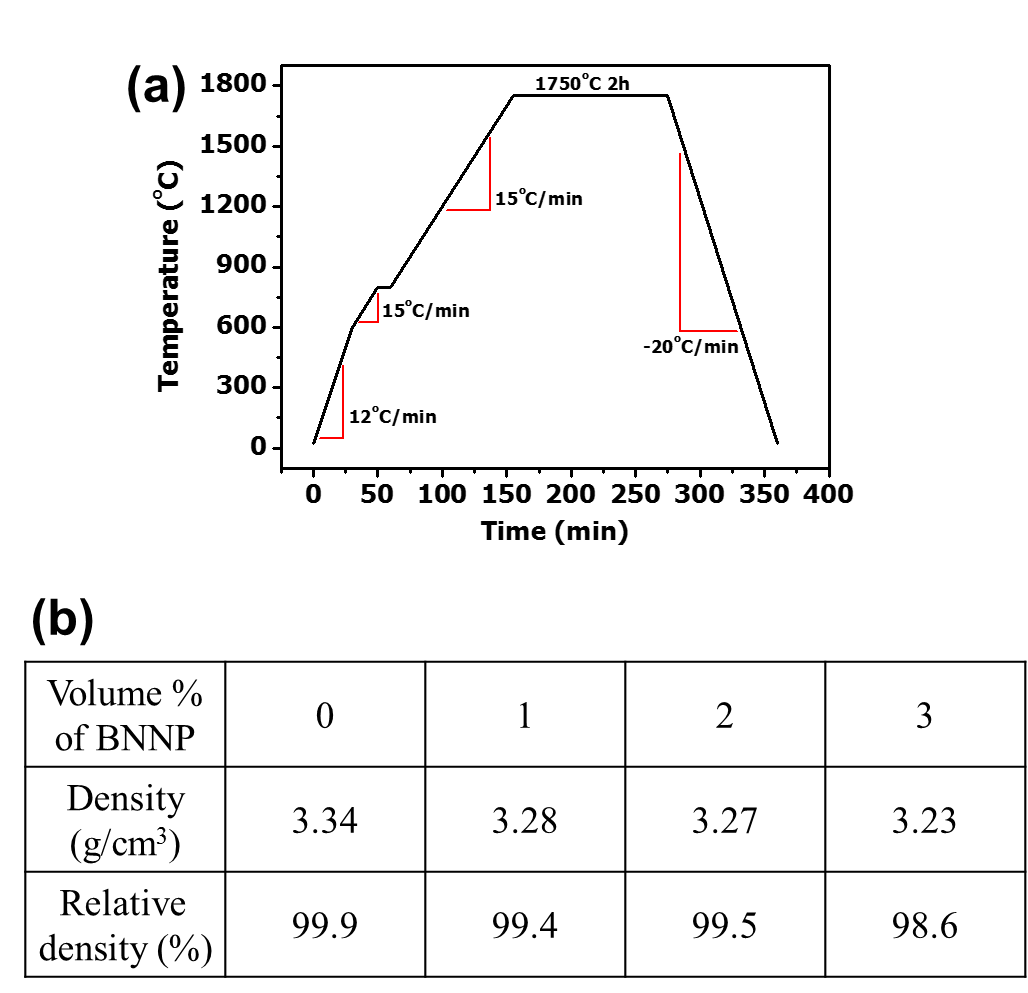


**Supplementary Figure 5. *Heating schedule and densification profile for hot pressing of BNNP/Si_3_N_4_ nanocomposites.***


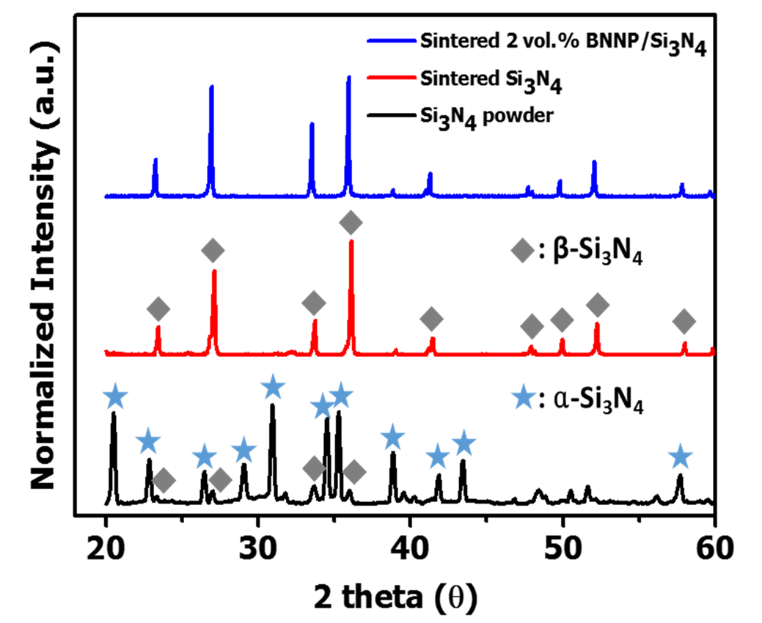


**Supplementary Figure 6. *XRD analysis of Si_3_N_4_ powders, sintered pure Si_3_N_4_ and 2 vol.% BNNP/Si_3_N_4_ nanocomposite.*** After hot pressing, alpha Si_3_N_4_ transformed into beta phase. Due to low contents of BNNPs, there is no characteristic peaks which indicate h-BN.


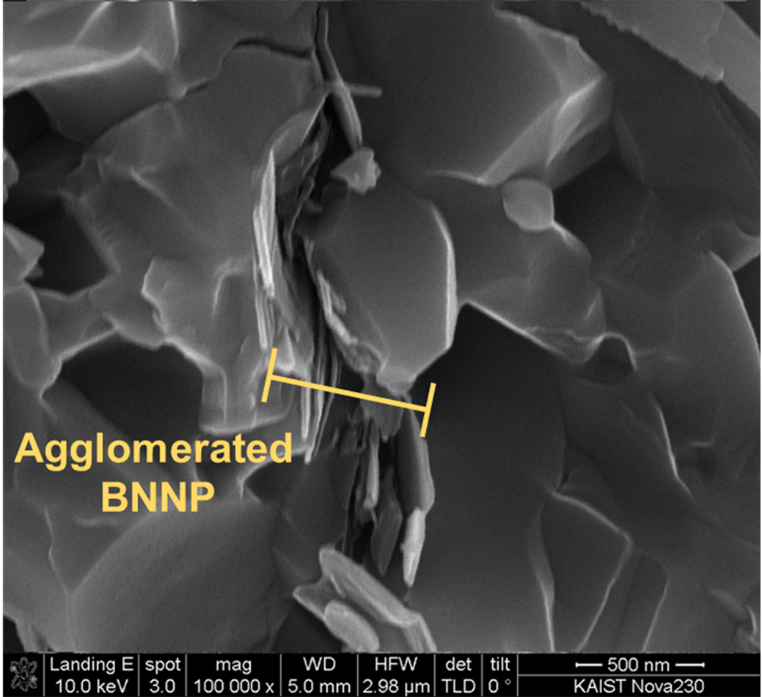


**Supplementary Figure 7. *SEM image of 3 vol.% BNNP/Si_3_N_4_ nanocomposite.*** BNNPs were agglomerated and formed the cleft in the matrix.


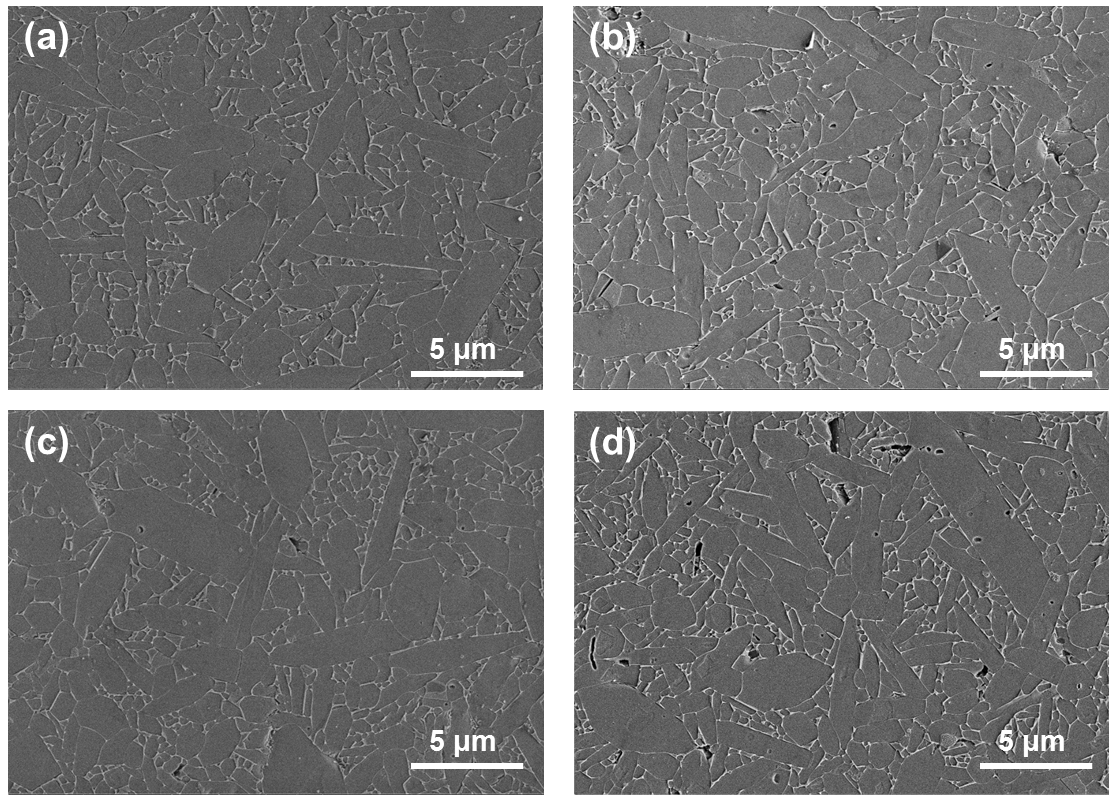


**Supplementary Figure 8. *Polished and etched surface of Si_3_N_4_ nanocomposites.*** (a) pure Si_3_N_4_, (b) 1 vol.% BNNP/Si_3_N_4_, (c) 2 vol.% BNNP/Si_3_N_4_, and (d) 3 vol.% BNNP/Si_3_N_4_

**Supplementary Figure 9. *Analysis of the grain size and shape of BNNP/Si_3_N_4_ composites.*** Blue line indicates average grain size of the composites, and red line shows average aspect ratio of the grains.


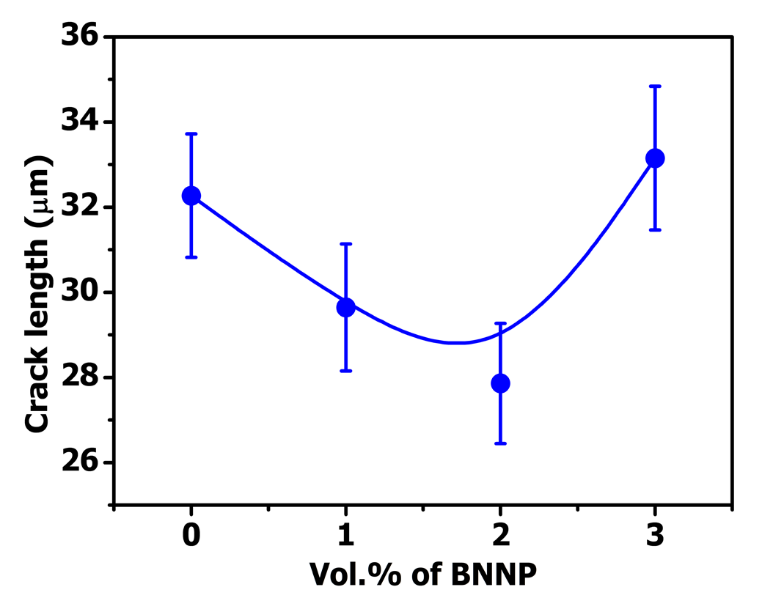


**Supplementary Figure 10. *Length of the crack which are conducted from the Vickers hardness test.*** Length of crack was measured by the SEM analysis.


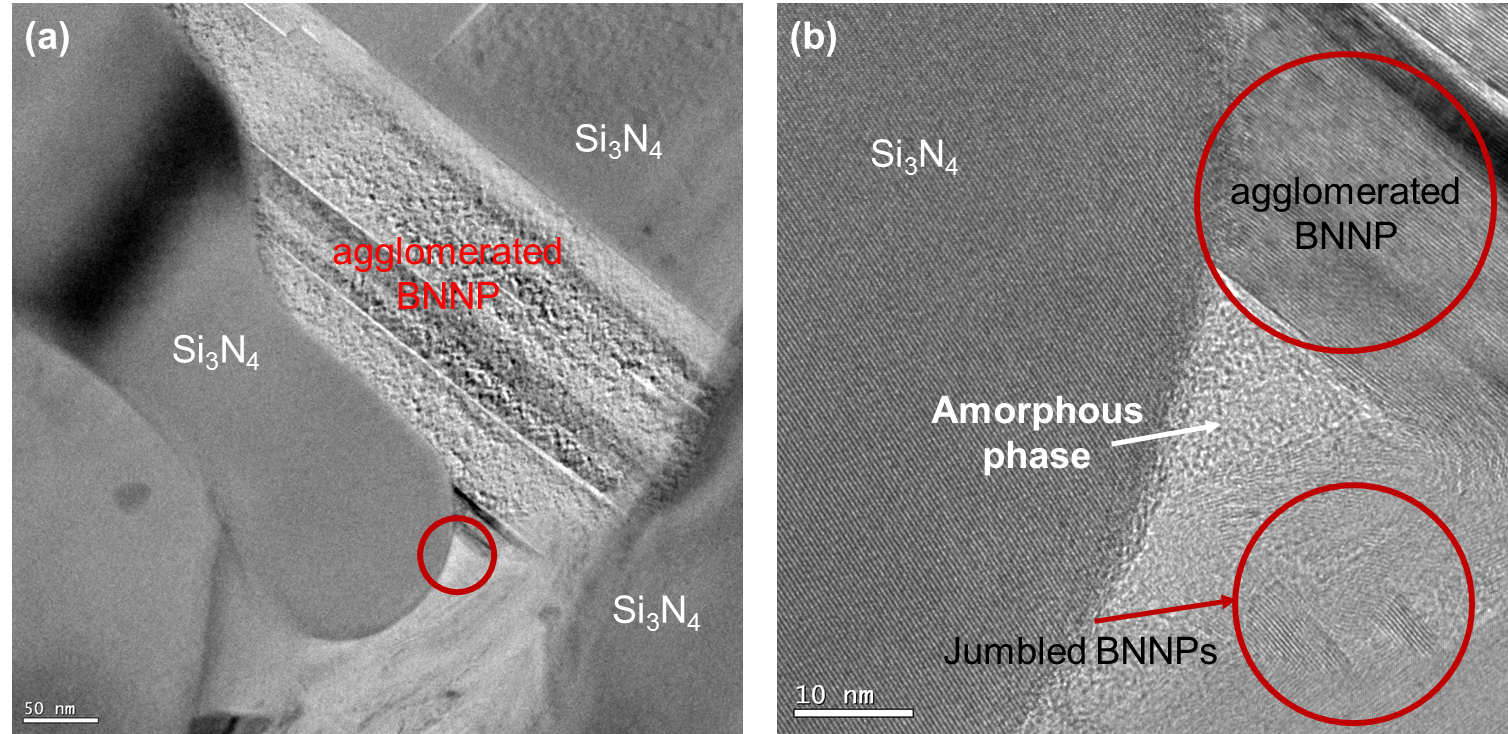


**Supplementary Figure 11. *TEM analysis of sintered 3 vol.% BNNP/Si_3_N nanocomposite.*** Amorphous phase, BNNP jumbled area, and agglomerated thick BNNP can be observed

***
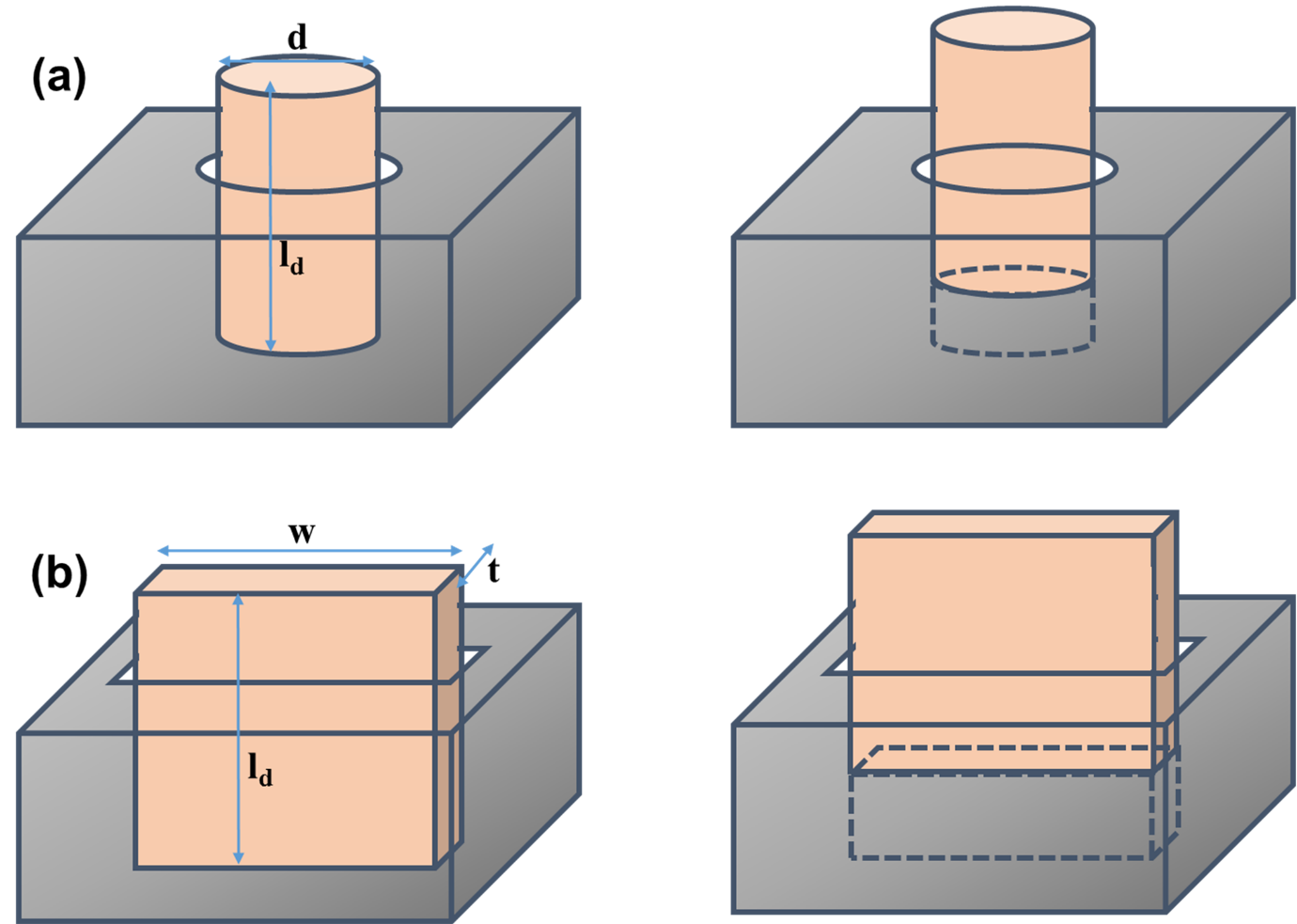
***

**Supplementary Figure 12. *Pull-out mechanisms in nanoplatelet reinforced ceramic matrix composites.*** The formulas are modified from fiber reinforced CMCs. If the nanoplatelet is assumed as square type, then l_d_ = w.


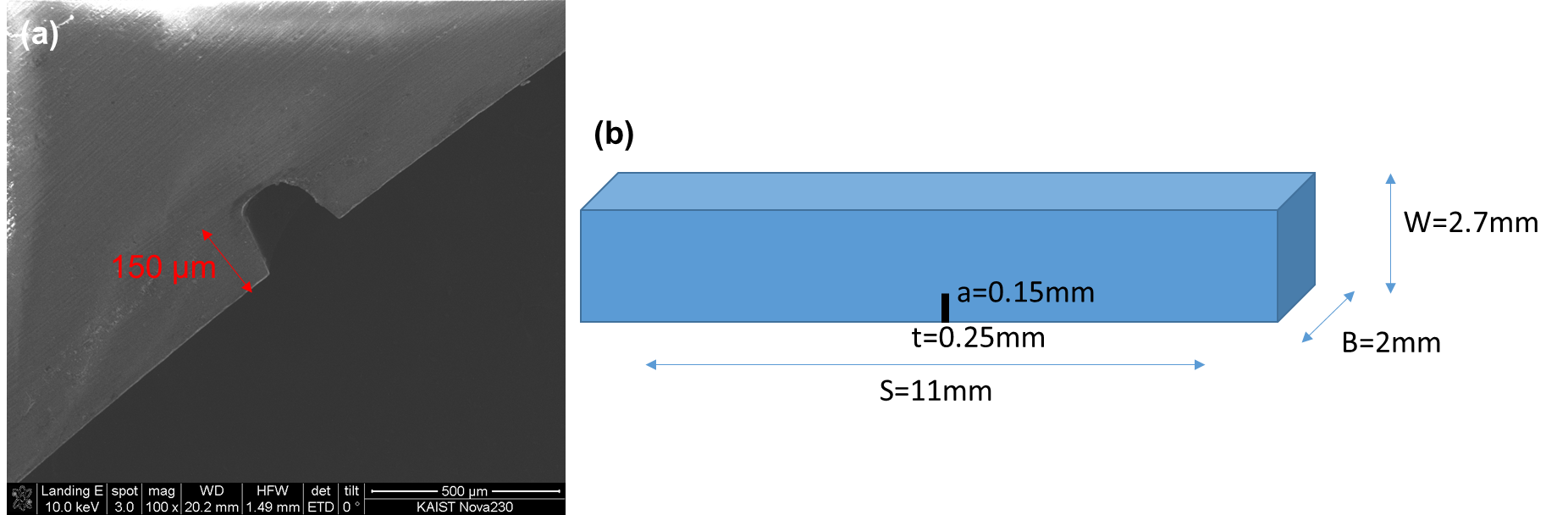


**Supplementary Figure 13. *Microstructure and schematic dimension of SENB test sample.*** The depth of notch was about 0.15 mm. The notch of the samples was created with a razor blade.

**Supplementary Table 1. *Densities of pure Si_3_N_4_ and BNNP/Si_3_N_4_ after hot pressing.*** Si_3_N_4_ sample contains 5 wt.% of Y_2_O_3_ and 2 wt.% of Al_2_O_3_


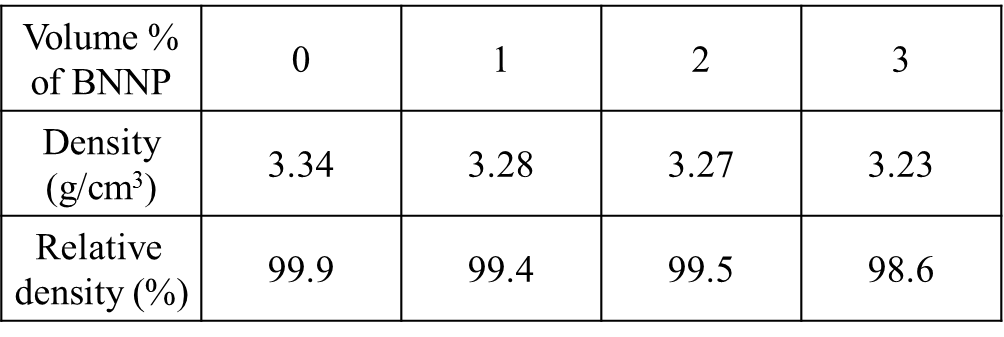


**Supplementary Table 2. *Hardness and toughness results of graphene-like nanomaterial reinforced ceramic matrix composites.*** Graphenes played a greater role in the increase in the hardness for the soft ceramic matrix.


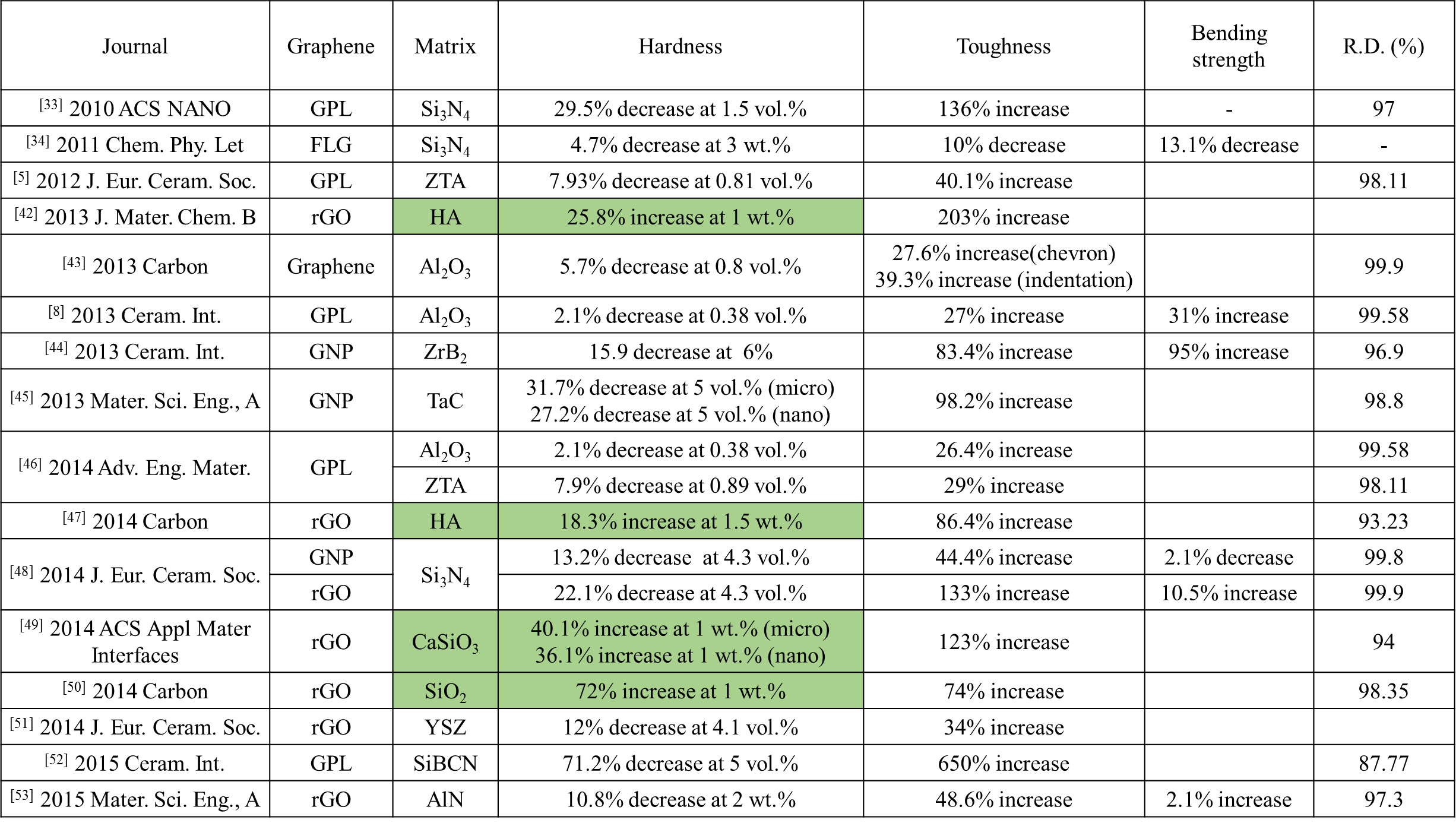


1. ) Corresponding author : Tel: (+82) 42 350 3812, E-mail: hojinryu@kaist.ac.kr (H. J. Ryu) [↑](#footnote-ref-2)
2. ) Corresponding author : Tel: (+82) 42 350 3327, E-mail: [shhong@kaist.ac.kr](mailto:shhong@kaist.ac.kr) (S. H. Hong) [↑](#footnote-ref-3)
